# Field Deployment of a Portable Optical Spectrometer for Methane Fugitive Emissions Monitoring on Oil and Gas Well Pads

**DOI:** 10.3390/s19122707

**Published:** 2019-06-16

**Authors:** Eric J. Zhang, Chu C. Teng, Theodore G. van Kessel, Levente Klein, Ramachandran Muralidhar, Gerard Wysocki, William M. J. Green

**Affiliations:** 1 IBM Thomas J. Watson Research Center, 1101 Kitchawan Road, Yorktown Heights, NY 10598, USA; tvk@us.ibm.com (T.G.v.K.); kleinl@us.ibm.com (L.K.); muralidr@us.ibm.com (R.M.); wgreen@us.ibm.com (W.M.J.G.); 2 Department of Electrical Engineering, Princeton University, Princeton, NJ 08544, USA; ccteng@princeton.edu (C.C.T.); gwysocki@princeton.edu (G.W.)

**Keywords:** absorption spectroscopy, infrared, diode laser, methane, natural gas, fugitive emissions, source estimation, angle-of-arrival, Gaussian plume model

## Abstract

We present field deployment results of a portable optical absorption spectrometer for localization and quantification of fugitive methane (CH_4_) emissions. Our near-infrared sensor targets the 2ν_3_ R(4) CH_4_ transition at 6057.1 cm^−1^ (1651 nm) via line-scanned tunable diode-laser absorption spectroscopy (TDLAS), with Allan deviation analysis yielding a normalized 2.0 ppmv∙Hz^−1/2^ sensitivity (4.5 × 10^−6^ Hz^−1/2^ noise-equivalent absorption) over 5 cm open-path length. Controlled CH_4_ leak experiments are performed at the METEC CSU engineering facility, where concurrent deployment of our TDLAS and a customized volatile organic compound (VOC) sensor demonstrates good linear correlation (R^2^ = 0.74) over high-flow (>60 SCFH) CH_4_ releases spanning 4.4 h. In conjunction with simultaneous wind velocity measurements, the leak angle-of-arrival (AOA) is ascertained via correlation of CH_4_ concentration and wind angle, demonstrating the efficacy of single-sensor line-of-sight (LOS) determination of leak sources. Source magnitude estimation based on a Gaussian plume model is demonstrated, with good correspondence (R^2^ = 0.74) between calculated and measured release rates.

## 1. Introduction

Natural gas (NG) harvesting has undergone accelerated growth due to increasing demand for alternative clean energy sources [1,2], with over half-million active wells for oil and gas extraction in the United States alone [3]. Methane (CH_4_), the primary constituent of NG, is of primary interest given its high energy density [4] and relatively clean combustion process [5], with modern applications [6,7,8] ranging from electricity generation to domestic heating and, more recently, as an automotive fuel (in the form of compressed NG). However, given the high radiative forcing [1] of CH_4_ (37× higher than CO_2_), its efficacy as a clean fuel is reliant on the suppression of fugitive emissions (up to 6.0% of total well production for conventional NG [2]), the majority of which occur during the NG production/extraction phase [1,2,9]. Ideally, point sensors with adequate CH_4_ sensitivity and molecular specificity should be located on-site for spatially resolved, continuous in situ leak monitoring [10]; however, the present lack of cost-effective CH_4_ monitoring solutions prevents large-scale implementation of source attribution technologies for the timely localization and quantification of NG leaks [11,12,13]. Presently, a spectrum of existing chemical sensing technologies of CH_4_ exist, although these methods [10,14] (e.g., chemiresistive, electrochemical, photoionization methods) are broadly susceptible to a wide array of volatile organic compounds (VOCs), thus presenting poor species discrimination [15] for leak magnitude estimation.

Infrared (IR) absorption spectroscopy has emerged in recent years as a promising solution for trace-gas detection which requires high levels of precision and molecular specificity [16,17,18], with applications ranging from health diagnostics [19,20] to environmental [21,22] and industrial process monitoring [23]. Fundamental rovibrational transitions in the mid-IR (3–25 µm) are readily targeted using quantum (or interband) cascade laser (QCL/ICL) technologies [24,25], while weaker overtone bands are typically measurable in the near-IR (NIR) using conventional tunable diode laser absorption spectroscopy (TDLAS) [17,26,27]. In particular, CH_4_ is accessible by the latter given the convenient presence of its 2ν_3_ harmonic overtones in the telecommunications U-band (1625–1675 nm) [27,28], and the spectrally resolved nature of TDLAS intrinsically enables excellent species discrimination through appropriate selection of interference-free transitions. In contrast to hyperspectral imaging [29] or non-dispersive IR techniques [16], TDLAS offers both significantly improved specificity and sensitivity, with noise-equivalent absorption (NEA) < 10^−5^ Hz^−1/2^ achievable in well-designed systems [16].

In this paper, we present a field deployable NIR TDLAS spectrometer for in situ CH_4_ monitoring on oil and gas well pads for localization and quantification of fugitive emissions. Outdoor testing is conducted at the Methane Emissions Technology Evaluation Center (METEC) site [30] at Colorado State University (CSU) using a variety of control and blind CH_4_ release experiments. Our portable near-IR TDLAS point sensor is based on a 5 cm open-path free-space design, and targets the CH_4_ R(4) overtone transition (6057.1 cm^−1^) to achieve single-ppmv-level sensitivity, and quantitatively demonstrates the performance attainable for CH_4_ fugitive emissions monitoring using only a single TDLAS sensor. In conjunction with wind velocity sensors deployed at METEC CSU, we demonstrate successful angle-of-arrival (AOA) leak source identification, and initial work will be presented using a classical Gaussian plume model for source magnitude estimation. We envision our source estimation methods to be widely applicable to a variety of sensing configurations (optical or otherwise), while providing a comparison benchmark for a next generation integrated photonic sensors with superior size, weight, power, and cost (SWaP-C) currently under development [18,27,31].

## 2. Sensor Design and Characterization

### 2.1. TDLAS Sensor Configuration

**Figure 1:**
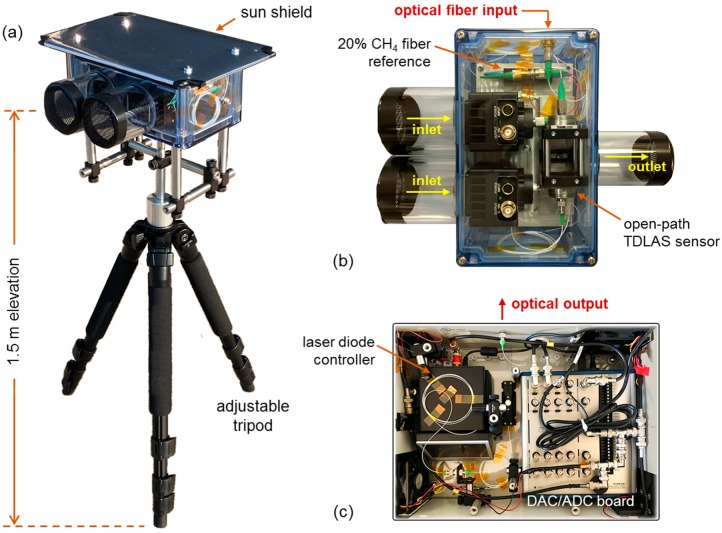
(**a**) The portable tunable diode-laser absorption spectroscopy (TDLAS) sensor mounted on a height-adjustable tripod (extensible up to 1.5 m). (**b**) Top view of the TDLAS sensor enclosure (V = 8 × 5 × 3 in.^3^). The gas inlets draw in ambient air via the photodetector TEC fans, and is probed for CH_4_ by the 5 cm open-path. (**c**) The TDLAS sensor control unit (V = 16 × 12 × 6 in.^3^), which houses the (DFB) laser diode, polarization controller, and DAC/ADC acquisition board.

The portable sensor unit is shown in Figure 1a, which consists of the TDLAS spectrometer mounted on top of a height-adjustable tripod (extensible up to 1.5 m) for manual elevation control. A reflective aluminum sunshield is mounted above the sensor unit to reduce heating from direct solar irradiance during extended field deployments. The physical construction of TDLAS optics is depicted in Figure 1b and comprises of a 5 cm open-path optical cell, two InGaAsP photodetectors (Model PDA10DT, Thorlabs, Newton, NJ, USA), and a 20 vol.% CH_4_ reference cell (3 cm) housed within a IP65-rated transparent polycarbonate enclosure (V = 8 × 5 × 3 in.^3^). The laser light at 1651 nm is input through an optical fiber feedthrough, which is split 90:10 via 1 × 2 fiber coupler for simultaneous sample (open-path) and reference measurement respectively. The open-path cell utilizes aspheric lens (Model PAF-X-7-C, Thorlabs, Newton, NJ, USA) collimators between the 5 cm free-space path, where each lens is alignment optimized and anti-reflection coated for etalon suppression (demonstrated by the relatively clean spectrum shown later in Figure 3b, Section 2.3). Directional ambient air flow through the TDLAS enclosure is aided by the photodetector thermoelectric cooler (TEC) fans, which draw air through the dual inlets (left) and through the open-path cell before ejecting at the outlet (right). Both inlet/outlet are sealed with coarse particulate filters to prevent accumulation of dust and contaminants within the TDLAS enclosure.

Figure 1c shows the acquisition and control electronics unit associated with the TDLAS sensor (similar to [21]), consisting of a distributed feedback (DFB) diode laser source and data acquisition (DAQ) card (Model NI-USB 6361, National Instruments, Austin, TX, USA) housed within a sealed IP66-rated polycarbonate enclosure (V = 16 × 12 × 6 in.^3^). The DFB diode laser selectively targets the 2ν_3_ R(4) CH_4_ transition [27,28,32] at 1651 nm (6057.1 cm^−1^), specifically utilized for immunity with respect to common atmospheric constituents (e.g., H_2_O, CO_2_, CO, and other VOCs of interest). The DFB laser is housed within an integrated TEC/current-driver unit (Model CLD1015, Thorlabs, Newton, NJ, USA) and provides up to 3 mW optical throughput (accounting for all insertion losses) into the TDLAS sensor at 1651 nm (*T_laser_* = 15 °C). The DFB laser is outcoupled from the enclosure via fiber feedthrough (top), directly leading to the input coupler on the TDLAS unit shown in Figure 1a. Data streaming (analog-to-digital conversion, ADC) and laser control (digital-to-analog, DAC) is accomplished through the DAQ board, which provides synchronous laser ramp and detector read-in (500 kHz channel sample rate) for real-time CH_4_ retrieval via serial data transfer for spectral analysis on a local computer.

In a nominal measurement configuration, the TDLAS sensor undergoes line-scanned measurements at a 500 Hz laser ramp rate, using a wavelength range spanning 6.5× the R(4) transition full-width half-maximum (FWHM~0.15 cm^−1^) to ensure that sufficient spectral baseline is acquired for normalization and accurate baseline removal. Given that the wavelength of the laser is not known a priori, the 3 cm CH_4_ reference cell serves primarily as a reference to ensure the DFB laser is correctly wavelength centered [32]. Additionally, the accuracy of TDLAS is particularly susceptible to detection baseline drifts [33] and, therefore, the baseline is measured (at 13% acquisition duty cycle) between each ramp sequence to ensure removal of transmission offsets in the measured spectra.

For typical real-time analysis, spectra at a specified interval (typically 1 s) are acquired and averaged, followed by application of a Voigt profile least-mean squares (VLMS) regression for CH_4_ retrieval. Due to the time required for spectral analysis (spectral averaging, baseline removal, and VLMS regression) the acquisition duty cycle is reduced (50%), resulting in a time resolution of 2 s per CH_4_ concentration point (although this is not an intrinsic limitation; parallelization of acquisition and analysis threads may bring this to full acquisition duty cycle without data loss [34]). In cases where higher time-resolutions are required, continuous streaming of 500 Hz ramp spectra may be acquired, followed by post-processing to achieve CH_4_ concentrations with time resolutions down to 2 ms. In this latter scenario, TDLAS becomes both computationally challenging and data-intensive due to the large number of acquired spectra and is, as in our case, solely utilized for sensor diagnostics (Section 2.2) rather than field measurements.

### 2.2. Sensitivity Analysis and Noise Characterization

**Figure 2:**
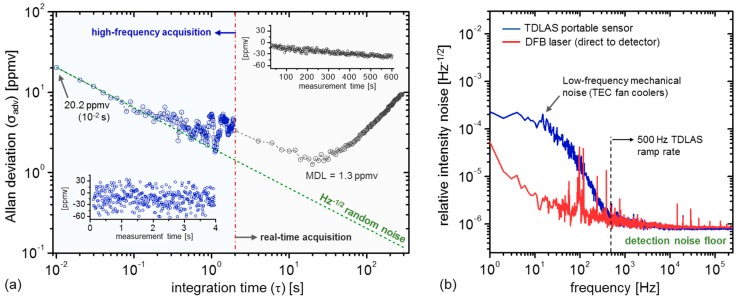
(**a**) Allan deviation analysis in two operating modes: (i) high-frequency acquisition (left, blue) and real-time acquisition (right, black), with CH_4_ time-series shown in the insets. The TDLAS sensor demonstrates short-term sensitivity of 20.2 ppmv at 10^−2^ s (noise-equivalent absorption (NEA) = 4.5 × 10^−6^ Hz^−1/2^), and a minimum detection limit (MDL) of 1.3 ppmv at 20 s. (**b**) The power normalized noise spectral density of the TDLAS sensor (blue), in comparison to the DFB laser source (red). The TDLAS line-scan frequency of 500 Hz is selected to mitigate the impact of low-frequency noise induced by the fan-cooled InGaAsP photodetectors.

Examples of CH_4_ concentration retrieval under high-frequency (high-time resolution) and real-time operating conditions are shown in Figure 2a (insets) in ambient laboratory conditions, where the former shows acquired CH_4_ time-series at 10 ms resolution (5-spectra averaging, 4 s total acquisition time), and the latter at 2 s (600 s acquisition). Allan deviation analysis [35] is used to quantify the sensitivity in both operating modes, demonstrating 20.2 ppmv (at 10^−2^ s) short-term precision (Figure 2a, blue), or NEA = 4.5 × 10^−6^ Hz^−1/2^, consistent with state-of-art TDLAS sensor systems [16]. Real-time sensitivity analysis (Figure 2a, black) yields a minimum detection limit (MDL) of 1.3 ppmv (at 20 s integration time), although ambient fluctuations during field deployment scenarios may cause significant stability time variation. To accommodate such stability time limitations during the field deployment, we utilize baseline removal techniques (described in further detail in Section 3.2 and depicted in Figure 5) to ensure that peak events are accurately captured. Given the 50% acquisition duty cycle of real-time analysis, we may theoretically expect a √2× sensitivity degradation factor with respect to ideal Gaussian noise (green line, Figure 2a); however, the larger deviation (2.3× from ideal at 2 s integration time) is likely due to the onset of short-term accuracy drift, as evidenced in the high-frequency Allan deviation near ~1 s.

Given a 5 cm measurement path length, a NEA of 4.5 × 10^−6^ Hz^−1/2^ for our TDLAS sensor corresponds to a minimum detectible absorption (MDA) coefficient *α_min_* = 8.9 × 10^−7^ cm^−1^∙Hz^−1/2^. The relatively short (5 cm) path length was selected as a compromise between alignment robustness for long-term field deployments and sensitivity requirements for CH_4_ leak detection, where sensors with sub-10 ppmv sensitivities yield diminishing returns for leak monitoring applications [36]. Additionally, our free-space TDLAS sensor benchmarks the performance attainable by a next-generation of integrated photonic chip sensors currently under development [18,27,31], which provide similar effective path lengths due to modal confinement limitations. These miniaturized chip sensors are intended to provide further benefits in size, weight, power, and cost (SWaP-C), and will be demonstrated in the next iteration of field testing. In the case where significantly enhanced sensitivities are desired, multi-path (or cavity-enhanced) designs along with mid-IR (quantum-cascade or interband-cascade laser) sources (targeting the fundamental ν_3_ band near 3.3 µm) may be integrated into the sensor unit to yield ppbv-level sensitivities [16].

Figure 2b shows the amplitude spectral density of optical throughput from the open-path cell (blue) compared against the DFB laser. The low-frequency noise contributions (<500 Hz) are due to the fan-cooled InGaAsP photodetectors housed within the TDLAS enclosure. This fan-induced noise is suppressed in our measurements by selection of TDLAS line-scanning rate of 500 Hz, which decouples the TDLAS spectral scans from the low-frequency noise floor [32]. Above 500 Hz, the intensity noise is dominated by the empirical detection noise floor (*σ_det_* = 8.0 × 10^−7^ Hz^−1/2^), yielding a calculated detection noise-equivalent power (NEP) of 7.2 × 10^−10^ W∙Hz^−1/2^ (at 100 kHz) given a total power of 0.9 mW through the free-space path (1.21 kV/W detector gain). In the ideal scenario where the system approaches this detection-noise limit, integration of the NEP spectral density over the measurement bandwidth yields a detection-limited MDA *α_det_* = 4.1 × 10^−7^ cm^−1^∙Hz^−1/2^, indicating that our TDLAS sensor operates at 2.2× above the empirical detection noise floor. Increasing the line-scan rate may reduce this factor by further reducing residual laser intensity noise, although at the cost of diminishing the spectral wavelength resolution during each scan sequence.

### 2.3. TDLAS Enclosure Residence Time and CH_4_ Spectra

**Figure 3:**
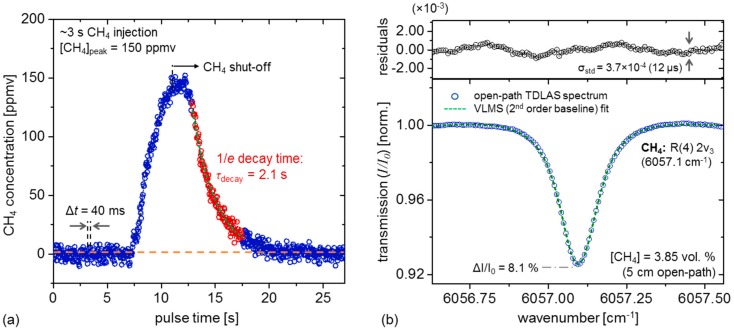
(**a**) Laboratory CH_4_ release from a 1.0 vol.% CH_4_ cylinder (air balance), measured in high resolution mode (40 ms time-resolution). The depicted peak is an average result of three consecutive peak-aligned measurements (~3 s CH_4_ release), with a 2.1 s characteristic 1/*e* decay time. The peak concentration attained (150 ppmv) is typical of leaks measured during the METEC field deployment. (**b**) A sample spectrum (2 ms) of a high-concentration CH_4_ release (3.85 vol.%) into the open-path cell, demonstrating the 2ν_3_ R(4) spectrum of CH_4_ at 6057.1 cm^−1^. A second-order polynomial is utilized during Voigt profile least-mean squares (VLMS) to accommodate the non-linear laser ramp and improve CH_4_ retrieval accuracy.

Figure 3a shows a controlled ~3 s CH_4_ release from a 1.0 vol.% cylinder (air balance) near the TDLAS sensor inlet to simulate a passing CH_4_ plume under laboratory conditions, yielding a peak concentration of 150 ppmv (typical of concentrations attained during field measurements). For display clarity, the time-series is depicted using 40 ms time-resolution (20 spectra averaging per CH_4_ point), and exponential modeling of the 90% to 10% peak level yields a characteristic flow response time of 2.1 s. Given a typical peak width of ≤20 s in typical field measurement scenarios, this response time is expected to accurately resolve temporal variations of CH_4_ peaks (i.e., the TDLAS chamber is expected to accurately reflect ambient concentrations). Furthermore, the real-time analysis interval of 2 s (Section 2.2) sufficiently captures changes on the order of the chamber response time and does not limit the temporal resolution of fast CH_4_ fluctuations.

A typical measured CH_4_ R(4) spectrum is depicted in Figure 3b for a high-concentration (3.85 vol.%) release. The spectrum is fit using a Voigt model [21,33], using line-shape parameters provided in the high-resolution transmission molecular absorption database [37] (HITRAN). The spectrum is acquired over 2 ms (500 Hz line-scan rate), with each spectral point averaged over 12 µs intervals for display clarity. In conjunction with VLMS regression, a quadratic term is utilized to accommodate the nonlinear laser ramp, yielding fractional absorption noise of σ_std_ = 3.7 × 10^−4^ (12 µs) as indicated by the standard deviation of the fitting residuals. It is notable that normalization to the line-shape FWHM (and accounting for the 15.5% duty cycle of the fit-significant window) yields an estimated MDA of 6.5 × 10^−7^ cm^−1^∙Hz^−1/2^, in reasonable agreement with the previously determined *α_min_* = 8.9 × 10^−7^ cm^−1^∙Hz^−1/2^. The minor discrepancy is likely a result of scan-to-scan variations of the TDLAS system that are not captured in a single-spectrum measurement.

## 3. Field Deployment Results

### 3.1. Sensor Deployment at the METEC Facility

**Figure 4:**
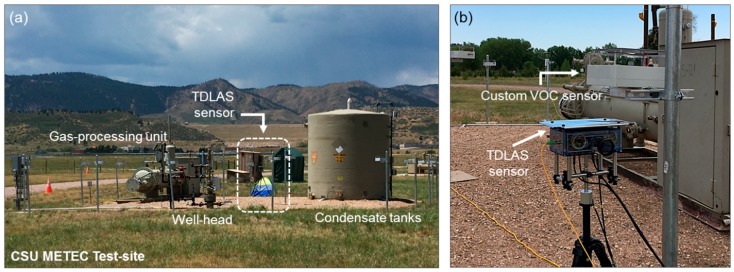
(**a**) Field deployment of the portable TDLAS sensor unit at the CSU METEC test-site. The site layouts typically include some combination of wellheads (or ‘trees’), gas-processing units (GPUs), and condensate tanks, all of which are possible sources from which leaks emanate. For this sample site layout (enumerated as Pad 1 in Table 1), the placement of the optical TDLAS sensor is shown in the white box. (**b**) Close-up of the optical sensor shown in (**a**). The TDLAS sensor is placed in close proximity to a customized metal oxide (MOX) sensor for accuracy benchmarking. The associated control and power units for the sensor are housed nearby the sensor assembly.

Controlled CH_4_ release experiments have been performed at the METEC CSU facility [30] based in Fort Collins, CO, taking place over a 5-day span during the third week of July. The deployment site consists of various well-pad configurations, offering an assortment of layout geometries based on wellheads, condensate tanks, and gas-processing units (GPUs) (shown in Figure 4a) with predefined leak points and source rates varying from 0 to 135 SCFH on each site. In conjunction with the TDLAS sensor, a customized chemiresistive metal oxide (MOX)-based VOC sensor (Model TGS2611, Figaro USA Inc, Arlington Heights, IL, USA) was placed in close proximity to validate our optical measurement results, as shown in Figure 4b. Although the MOX sensor is expected to lack discrimination capabilities between CH_4_ and other VOCs [14,15], the leak experiments are based on a well calibrated natural gas mixture (85% CH_4_, with minor remaining constituents 10.2% C_2_H_6_, 0.7% C_3_H_8_, remainder N_2_ and CO_2_), thus serving as a reasonable benchmark against which our TDLAS measurement may be compared. Additionally, an ultrasonic anemometer (Model 81000, R. M. Young Co., Traverse City, MI, USA) is deployed on the test-site for concurrent wind-velocity measurements, which is used for leak angle-of-arrival (AOA) detection of the source-to-detector line-of-sight (LOS) via concentration weighted wind-angle localization, as well as Gaussian plume (Section 3.4) and machine-learning regression models [38] for source magnitude estimation.

Over the duration of the 5-day deployment [21], a total of 16.6 h of CH_4_ measurements were acquired using the TDLAS sensor (along with MOX sensor and wind velocity data), consisting of a combination of control (4.4 h) and blind (12.2 h) release experiments. In the former (control) case, the TDLAS sensor inlet is appropriately oriented towards a known source for direct LOS CH_4_ leak detection and is used for accuracy benchmarking against MOX sensor measurements (as shown later in Figure 7, Section 3.2), where emissions span relatively high flow-rates ranging from 68 SCFH to 135 SCFH to ensure that a multitude of CH_4_ peaks may be used for TDLAS and MOX sensor correlation. In the latter (blind) experiments, substantially visible peaks were available on only 1.9 h of measurements due to spatial coverage limitations intrinsic to single sensor measurements (i.e., the placement of the TDLAS sensor was not always aligned downwind of the leak source). The blind releases were typically of smaller magnitude (<40 SCFH) and have been incorporated into the models in Section 3.4 for source estimation of lower flow rates. Table 1 summarizes the compilation of 5 control and 2 blind leak experiments, along with the calculated AOA (or LOS leak angle, based on the method described in Section 3.3). In general, we note that the calculated leak-angle deviation is larger in the case of blind experiments, which is attributable to the smaller leak-rates (<40 SCFH) utilized in those cases. Furthermore, it is clear by comparison of the average wind-velocity and leak location columns in Table 1 that, in all cases, the sensor is downwind. The remaining blind experiments have not been displayed as no substantially visible peaks were observed, due to the poor alignment of the wind with the TDLAS sensor LOS; therefore, no meaningful AOA could be calculated in those cases.

### 3.2. CH_4_ Leak Data Acquisition and Processing

**Figure 5:**
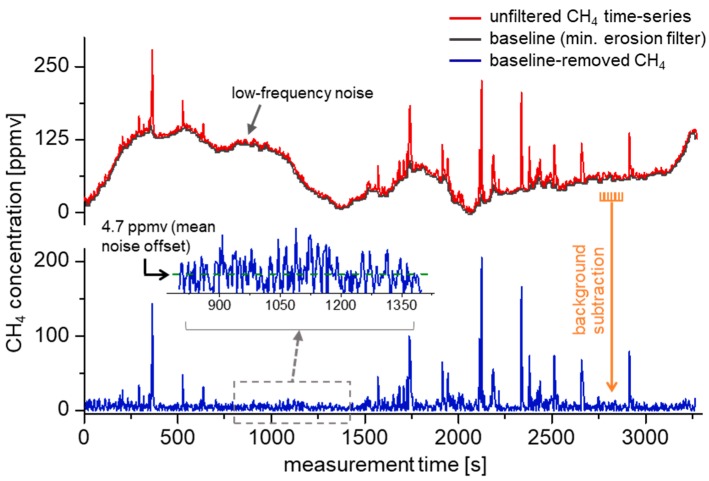
Removal of the TDLAS sensor baseline via local minimum erosion [39]. Concentration drifts are primarily due to spectral baseline fluctuations in the presence of large ambient temperature variations. The erosion filter is applied at 20 s intervals to identify and null the background (black curve). A side effect of this method involves the presence of a baseline offset (inset), which is removed via peak thresholding prior to AOA determination.

A typical CH_4_ acquisition sequence is shown in Figure 5 (representing Blind 1), which depicts the raw retrieved CH_4_ concentration (red), along with the baseline-removed time-series (blue) via a local minimum erosion filter to increase contrast visibility for peak event detection [39]. The concentrations are calculated using VLMS regression (Section 2.3), where each concentration point comprises an average of 500 spectra such as that shown in Figure 3b and is acquired every 2 s (50% acquisition duty-cycle, followed by spectral fitting).

A thermistor housed within the MOX sensor unit simultaneously tracks the temperature during acquisition of TDLAS data, and the average temperature over the span of each experiment is used to correct the absorption strength [28,37]:

(1) $$S_{\eta \to \eta \prime }(T)=S_{\eta \to \eta \prime }(T_{ref})\cdot \frac{Q(T_{ref})}{Q(T)}\cdot \frac{\exp(-c_{2}\cdot E_{\eta }/T)}{\exp(-c_{2}\cdot E_{\eta }/T_{ref})}\cdot \frac{1-\exp(-c_{2}\cdot \nu_{\eta \to \eta \prime }/T)}{1-\exp(-c_{2}\cdot \nu_{\eta \to \eta \prime }/T_{ref})}$$

(2) $$\delta \nu_{L}(p,T)={\left(\frac{T_{ref}}{T}\right)}^{n}\cdot \left[\gamma_{air}(p_{ref},T_{ref})\cdot (p-p_{s})+\gamma_{self}(p_{ref},T_{ref})\cdot p_{s}\right]$$

Equation (1) provides the temperature correction to the CH_4_ R(4) transition line-strength *S_η→η_*_’_(*T*) due to lower-state population depletion, which accounts for the temperature-dependent impact of the internal partition function *Q*(*T*), Boltzmann population factor, and stimulated emission. The temperature and its reference standard are denoted by *T* and *T_ref_* = 296 K respectively, *p_ref_* = 760 torr, *c*_2_ = 1.439 cm∙K is the second-radiation constant, *E_η_* = 104.8 cm^−1^ is the lower-state transition energy, and ν*_η→η_*_’_ is the center wavenumber (6057.1 cm^−1^) of all transitions constituting the R(4) line [37]. Equation (2) defines the impact of the air-broadening temperature coefficient *n* (where *n* = 0.72), which has been applied equally to both the air (*γ_air_*) and self (*γ_self_*)-broadening coefficients (a reasonable assumption given the typically small trace-gas sample partial pressures *p_s_* relative to the ambient pressure *p*). Given a typical CH_4_ concentration of 100 ppmv at 310 K, the above considerations introduce a line-strength correction factor of 4.7% and Lorentz pressure-broadening variation of 3.3%, which contributes to a peak absorption decrease of 6.5% relative to STP conditions and must be accounted for to ensure accuracy of CH_4_ retrieval. In the present case, the average temperature of a single acquisition sequence has been used for temperature correction of the absorption strength; however, this does not account for real-time thermal variations within the acquisition sequence, which may occur as a result of wind conditions, cloud cover, etc. In future deployments, further accuracy improvement will be gained by performing real-time temperature correction using a thermal reading within the TDLAS enclosure to ensure that in situ corrections of line-strength and air-broadening via Equations (1) and (2) are appropriately accounted for.

In addition to thermal correction factors, a significant deviation from atmospheric pressure occurs at the high altitude of the METEC facility (located in Fort Collins, CO, USA), and barometric reading averages over the duration of experimental data in Table 1 yields an ambient pressure of 843.9 mbar (633.0 torr), which is used to account for the pressure-shift [37] of the transition centers relative to the CH_4_ reference cell (740 torr). In comparison to STP conditions, line-shape simulations yield an absorption minima shift of 6.0 × 10^−4^ cm^−1^ which is 0.4% of the CH_4_ R(4) FWHM under the conditions of our field deployment.

Due to significant temperature drifts over the course of each experiment, concentration fluctuations due to laser ramp nonlinearities and Fabry–Pérot etalon drifts dominate low-frequency CH_4_ variations (visible in the red curve of Figure 5). The magnitude of these variations scale with respect to path length and transition line-strength (in accordance with the Beer–Lambert law [16,33]), and its significant amplitude is attributable to the small 5 cm optical path length and near-IR overtone detection, whose line-strengths are over 10^2^× weaker than the strongest ν_3_ fundamental transitions. Despite the large magnitude of these baseline variations, the frequency is highly decoupled from characteristic CH_4_ peak events, which occur on the order of the TDLAS sensor stability time (~20 s). Various methods exist for the thresholding of peak events [39,40]; in our case, a simple local minimum erosion [39] is utilized, which involves taking the baseline as the smallest value in a 20 s time-series bin. This effectively suppresses the baseline without substantially attenuating the CH_4_ peaks, albeit with an intrinsic noise offset (Figure 5, inset). In order to distinguish peak events from this noise offset, only peaks with averages above a defined threshold (~12 ppmv) are considered and will be discussed in Section 3.3.

**Figure 6:**
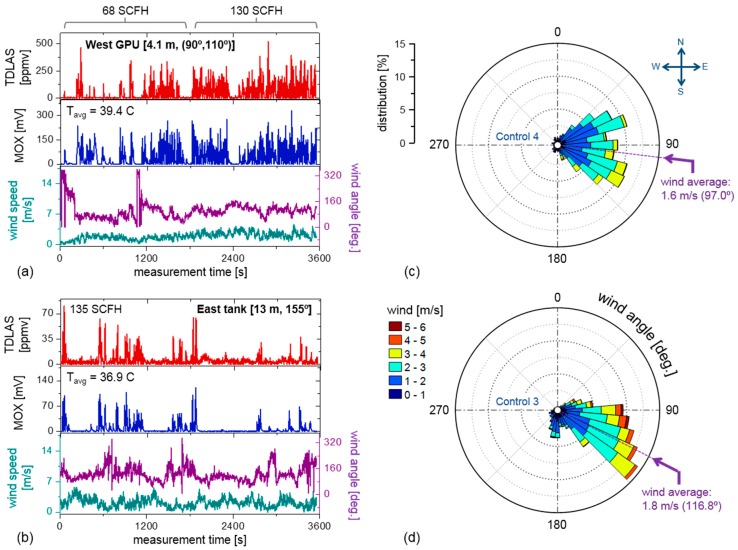
(**a**) Concurrent measurements of CH_4_ from the TDLAS, MOX, and anemometer data for Control 4. General visual agreement is observed between the TDLAS and MOX sensors. The average temperature of 39.4 °C was acquired using an internal thermistor in the MOX sensor unit. (**b**) Similar results for Control 3. (**c**,**d**) depict the corresponding wind-rose of the anemometer data. The results of AOA retrieval for these experiments are shown later in Figure 8, Section 3.3.

Figure 6a,b show results using the above analysis for two experiments (Control 4 and 3 respectively). MOX sensor and anemometer data are concurrently acquired with the TDLAS unit, and the resulting time-synchronized results (red: TDLAS; blue: MOX; purple/green: anemometer) demonstrate excellent visual correspondence between the MOX and TDLAS time-series. From Figure 6a (Control 4), we qualitatively note greater density of peaks occurring in the high flow-rate case, where the experiment is performed successively at two flow-rates (68 SCFH and 130 SCFH) over the span of 3552 s, whilst maintaining the TDLAS sensor 4.1 m west of the GPU leak source. Figure 6b (Control 3) shows the results of a uniform 135 SCFH control leak on the thief hatch of the condensate tank, with the sensor placed 13 m downwind (116.8°, southeasterly wind). Lower CH_4_ peak concentrations are observed (and expected) given the 4.7 m elevation of the thief hatch leak point in addition to the greater sensor-to-leak distance than that shown in Figure 6a (Control 4). Similar analyses have been performed for all control and blind experiments, and the resulting leak AOA calculations are summarized in Table 1. Figure 6c,d are wind-rose graphs corresponding to anemometer data in 6a,b. In both cases, the leak is observed in a general upwind orientation from the TDLAS/MOX sensor (Table 1), as expected in the simple case where a CH_4_ leak plume macroscopically proceeds along the wind direction. It is useful to note that by meteorological convention [41], a northerly wind (i.e., wind angle 0°) indicates that wind is blowing north to south, which is the standard adopted throughout this paper.

**Figure 7:**
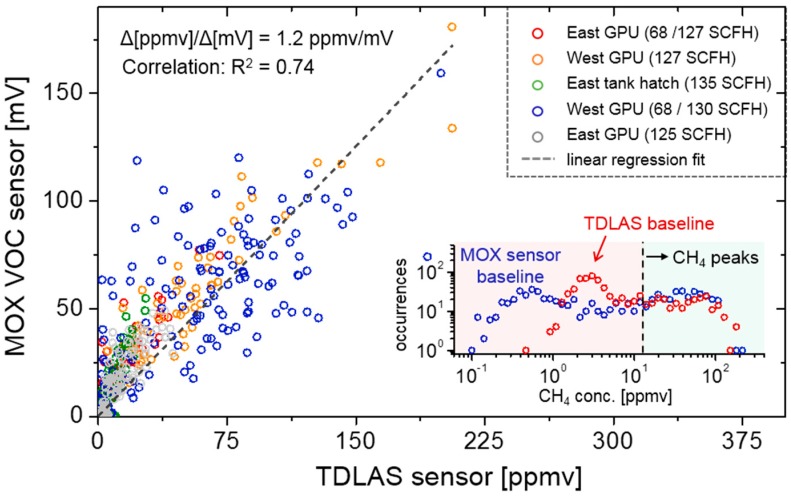
Correlation between TDLAS sensor readings and a proximally located MOX sensor during the 5 control leak measurements at varying locations and leak rates. The data spans a total of 4.4 h with 20 s integration time per point, yielding a scaling factor of 1.2 ppmv/mV. The inset shows a scatter histogram plot, indicating the distribution of baseline noise dominates CH_4_ measurements below 12 ppmv and defines the threshold for peak discrimination.

To quantitatively assess the agreement between the TDLAS and MOX sensors, their correlation (using 20 s integration bins) is displayed in Figure 7 for each of the 5 control experiments from Table 1. Linear regression yields a slope of 1.2 ppmv/mV, with correlation R^2^ = 0.74. Although the TDLAS and MOX sensor units are placed in close proximity, possible causes of correlation deviations from linearity include MOX sensor hysteresis and response time, which cause peak ‘pile-up’ during dense CH_4_ plumes from large or nearby leaks, as well as the directional flow orientation of the TDLAS sensor, which results in peak attenuation if the wind angle is not within the source-detector LOS. To avoid this asymmetric flow limitation in future deployments, an isotropic flow chamber is under development to eliminate directional selectivity of ambient CH_4_ detection.

To visualize the distribution of signal and noise of the CH_4_ time-series measured by both TDLAS and MOX sensors, the inset of Figure 7 shows a histogram of all 20 s time bins from each control experiment in the correlation plot. Both the TDLAS and MOX sensor data distributions are shown, with the latter scaled to parts-per-million (by volume) units using the correlation slope of 1.2 ppmv/mV. When expanded to a logarithmic scale, convergence of the peak distributions between the sensors agree only above a threshold of [CH_4_]_thresh_ = 12 ppmv, below which the CH_4_ concentrations (averaged to 20 s) are dominated by the intrinsic noise offset of the baseline erosion filter. Visible noise ‘peaks’ or distribution maxima of 3 ppmv and 0.6 ppmv are observed for the TDLAS and MOX sensors respectively, where the former is in reasonable agreement with the previously measured signal floor in Figure 5 (inset). The use of local minimum erosion (at 20 s bins) entails a noise offset similar to the nominally estimated Allan deviation analysis in Figure 2a and requires thresholding to distinguish true CH_4_ peaks from the noise floor.

### 3.3. CH_4_ Angle-of-Arrival (AOA) Source Determination

Based on the CH_4_ and anemometer data shown in Figure 6a,b, the corresponding AOA is calculated based on the mean wind angle weighted by CH_4_ concentration, where only peaks above a threshold value of [CH_4_]_thresh_ are utilized (Figure 7, inset) to mitigate noise in the AOA calculation. The time-series are binned into 20 s averaging periods, given that measured CH_4_ peaks are typically on the same order. Heuristically, the selection of a 20 s characteristic time is long enough to mitigate misalignment of wind and CH_4_ peak events (due to the TDLAS sensor response time), and sufficiently short to avoid averaging signals down to the noise floor. An average of the measured wind angle weighted by the CH_4_ peaks [42] yields a simple measure of AOA (denoted as <α_AOA_>) and deviation δα_AOA_:

(3) $$\langle \alpha_{AOA}\rangle =\frac{\sum_{i}\omega_{i}\cdot \varphi_{i}}{\sum_{i}\omega_{i}}$$

(4) $${\delta \alpha_{AOA}}^{2}=\frac{{\sum_{i}\omega_{i}\cdot \left(\varphi_{i}-\langle \alpha_{AOA}\rangle \right)}^{2}}{\sum_{i}\omega_{i}-1}$$

**Figure 8:**
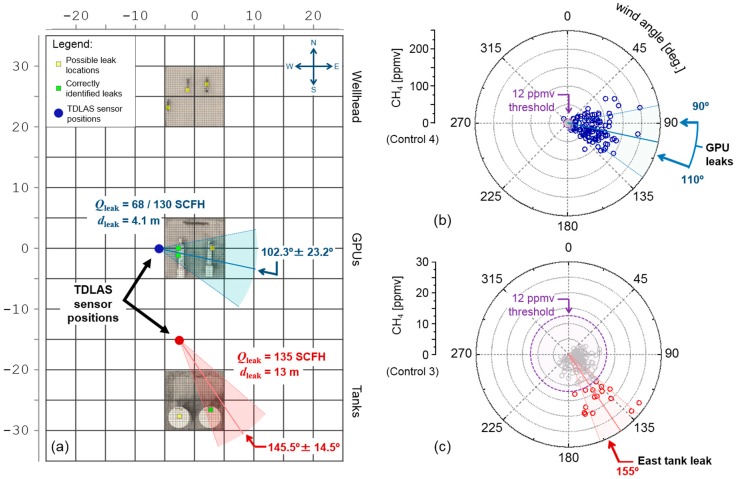
(**a**) Site layout for the experiments shown in Figure 6 (horizontal/vertical scale bar in meters). The source angle for the two depicted release experiments (blue and red) are determined using a concentration weighted average wind angle, shown in (**b**) and (**c**) respectively. The errors are correspondingly calculated using a weighted variance of wind angles from the mean. To ensure accurate AOA retrieval, signal thresholding ([CH_4_]_thresh_ = 12 ppmv) was used to eliminate erroneous peaks resulting from noise offsets in the CH_4_ baseline.

Equation (3) describes the AOA as a weighted average of wind angle *φ_i_* (accounting for the cyclic nature of *φ_i_* at null wind angle [42]) with weight factors *ω_i_*, which in the results displayed in Table 1 and Figure 8 is simply the CH_4_ concentration, i.e., *ω_i_* = [CH_4_](*t_i_*). In more advanced physical models, it may be necessary to model *ω_i_* as a function of wind speed, instantaneous wind angle, and/or leak distance; however, the simple model in our experiments are sufficient to yield reasonable AOA determination. An unbiased estimate of the leak angle variance is provided by Equation (4), which includes the effect of Bessel’s correction in the denominator given the sampling of CH_4_ peaks over the span of an experiment. Together, Equations (3) and (4) yield complete information <α_AOA_> ± δα_AOA_ of the leak angle as determined by a single TDLAS sensor, which corresponds to the results shown in Table 1.

Visualizations of the above calculations are depicted in Figure 8b,c, corresponding to the time-series datasets from Figure 6 (Control 4 and 3, displayed respectively as blue and red). The gray points indicate all CH_4_ concentration values below [CH_4_]_thresh_, which are primarily dominated by the noise floor and are discarded prior to AOA calculations. The resulting angles <α_AOA_> ± δα_AOA_ are displayed in the red and blue shaded regions and are marked with respect to their physical locations on the well pad (Figure 8a). Control 3 (Figure 8c, red) arises from a thief hatch leak on the east condensate tank, yielding calculated leak angle of 145.5 ± 14.5°, consistent with an expected angle of 155°. During Control 4 (Figure 8b, blue), the leak emissions simultaneously arise from equally split flows from dual valve release points on the west GPU, which result in a larger spread (102.3 ± 23.2°) to encompass both leak directions (90° and 110°) from each valve. The potential (yellow) and successfully identified leak points (green) are displayed in Figure 8a, and comparison with Figure 6c,d demonstrate the general downwind placement of the TDLAS sensor, where the average wind directions for Controls 3 and 4 occur at 116.8° and 97.0°, respectively.

The use of a single TDLAS sensor intrinsically allows only determination of leak angle, which in the example of Control 4 (blue), does not enable discrimination between multiple leak sources within the estimated AOA [43] (i.e., both the east and west GPUs are potential leak candidates, although only the west GPU is the culprit). In this case, a minimum of two sensors are necessary to ascertain an enclosed region from which a leak emanates from, which becomes progressively more precise as the number of sensors increase (or the source-detector distance decreases). In practical measurement scenarios, however, it may be merely sufficient to utilize a single appropriately placed sensor to ensure that leaks from each potential emission point occurs along a unique AOA, thus allowing accurate localization from long-term measurements.

### 3.4. CH_4_ Source Magnitude Estimation Models

A variety of methods exist for forward plume dispersion modeling from a point source under defined wind conditions [44], based on Gaussian, Lagrangian, and/or computational fluid dynamics (CFD)-based models which accurately predict CH_4_ concentrations given a defined sensor placement. However, the full inversion problem (i.e., determining the source magnitude from downwind CH_4_ concentrations), poses a significant challenge [45,46] given the computational complexity and limited spatial information available from a single TDLAS point sensor. Probabilistic methods (e.g., recursive Bayesian inferencing) are of limited efficacy in the present case, given plume model parametrization uncertainty and stochastic error inherent in the short-term (<1 h) CH_4_ peak measurements [47]. Despite the shortcomings of our relatively small dataset size, we may nevertheless apply a simplified Gaussian plume model heuristic (described below) to obtain physical insight into the emission process, which may be generally applied to emission datasets extending beyond our limited tests.

Our quantification methodology begins with the assumption of steady-state conditions where advective transport dominates in the downwind direction (*x*). The Gaussian plume model [44,45] provides a measure of the concentration at a location ***r*** = (*x*,*y*,*z*) as measured by the TDLAS point sensor, given a point source with a known (i.e., measured) emission rate *Q_m_*:

(5) $$\left[CH_{4}](r)=\frac{Q_{m}}{2\pi \cdot \langle \left|v_{w}\right|\rangle \cdot \sigma_{y}\cdot \sigma_{z}}\cdot \exp\left[-\frac{y^{2}}{{2\sigma_{y}}^{2}}\right]\cdot \zeta (z,H,\sigma_{z}\right)$$

(6) $$\zeta (z,H,\sigma_{z})=\left\{\exp\left[-\frac{{\left(z-H\right)}^{2}}{{2\sigma_{z}}^{2}}\right]+\exp\left[-\frac{{\left(z+H\right)}^{2}}{{2\sigma_{z}}^{2}}\right]\right\}$$

where 〈|***v_w_***|〉 is the average wind speed, *H* is the emission height, and *σ_y_*, *σ_z_* denote the lateral and vertical spread in the plane perpendicular to the wind direction (*x*). The exponential terms on the right-hand side of Equation (5) arise from plume spreading, and the vertical plume dispersion term ζ(*z*,*H*,*σ_z_*) defined in Equation (6) includes the impact of ground reflection to ensure conservation of mass [45]. Intuitively, the presence of <|***v_w_***|> accounts for the dilution of the measured concentration through advection transport (i.e., greater wind speeds result in dilution along the downwind direction), while the effect of the downwind location *x* is implicitly given by the *σ_y_*(*x*) and *σ_z_*(*x*) dependence, which provides a measure of the rate of plume dispersion. Based on a single-particle Lagrangian dispersion model, it is well known [45,48] that *σ*~*x^n^*, where *n* = 1 for length scales below Lagrange integral distance *D_L_* (i.e., *x* << *D_L_*), while *n* = ½ for *x* >> *D_L_*, with *D_L_*~100 m for relatively unstable wind conditions as encountered in our release experiments [45]. The interpretation of *D_L_* is the crossover distance between which plume spread may be modeled via ballistic or eddy-diffusive transport, where the former case of ballistic transport (*x* << *D_L_*) is relevant for distance scales presently under consideration. The general approach utilized here is the identification of a ‘characteristic’ downwind peak height, which may be used to model *Q_c_* given known location ***r_s_*** of the TDLAS point sensor with respect to the upstream emission location.

**Figure 9:**
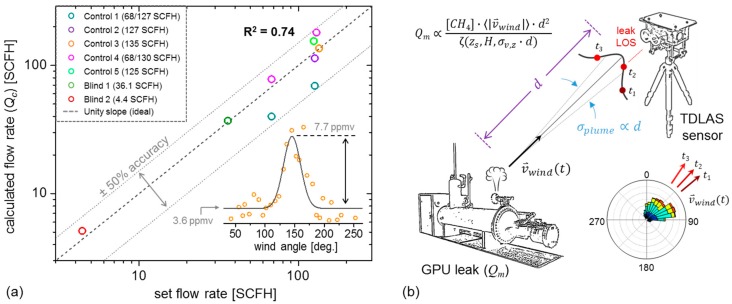
(**a**) Demonstration of the source emission magnitude consistency with the Gaussian plume model. The ideal linearity is given by the gray line (unity slope, dashed), with the 50% accuracy bounds (dotted lines). The inset shows an example of the measured Gaussian profile and its corresponding fit (Control 3) measured across a range of wind angles. (**b**) Pictorial demonstration of the Gaussian profile measurement. As the wind velocity vector varies (*t*_1_ to *t*_3_), the TDLAS sensor uses a single line-of-sight (LOS) to probe different parts of the plume.

Figure 9b depicts the general layout of a leak emission point with TDLAS sensor placed at some downwind distance *d*. Given that the plume profile is assumed to take a Gaussian form, each measured CH_4_ peak acquired at some time (e.g., time *t*_1_) probes only a particular point on the profile, while the variation of wind vector (e.g., at time *t*_2_, *t*_3_, etc.) ensures the aggregate peaks acquired over some duration effectively maps the shape of the Gaussian plume for a fixed sensor vertical displacement *z_s_* (assuming negligible vertical wind shear). An example is shown in the inset of Figure 9a for Control 3, from which a Gaussian regression fit (assuming to first order the negligible contribution of ground plume reflections) yields a characteristic downwind CH_4_ peak height of [CH_4_](***r_s_***) = 7.7 ppmv (i.e., a concentration peak measured at the detector along the leak LOS: ***r_s_*** = (*d*,0,*z_s_*), where *x* = *d* is the sensor downwind distance, *y* = 0 indicates measurement along the AOA with no lateral deviation, and *z* = *z_s_* is the sensor height). Under conditions where *d* << *D_L_*, we may parametrize *σ_y_* and *σ_y_* using the relations *σ_y_*(*d*) ≡ *σ_v,y_*∙*d* and *σ_z_*(*d*) ≡ *σ_v,z_*∙*d*, such that for *y* = 0, we may rearrange Equation (5) to obtain an estimated calculation *Q_c_* of the true flow rate *Q_m_* based on the retrieved [CH_4_](***r_s_***):

(7) $$Q_{c}=\kappa \cdot (2\pi \cdot \sigma_{v,y}\cdot \sigma_{v,z})\cdot \frac{[CH_{4}](r_{s})\cdot \langle \left|v_{w}\right|\rangle \cdot d^{2}}{\zeta (z_{s},H,\sigma_{v,z}\cdot d)}$$

In Equation (7), κ has been introduced as a heuristic dispersion modifier to account for measurement non-idealities (e.g., plume rise, near-ground measurements, low 〈|***v_w_***|〉 etc.), and is empirically calibrated by comparison of *Q_c_* to the known leak rates *Q_m_* (Table 1). Figure 9a demonstrates the results of Equation (7) applied to the data in Table 1, using a TDLAS sensor elevation *z_s_* = 1.5 m, and condensate tank heights of *H* = 4.7 m and 3.5 m respectively used in Control 3 and Blind 1, while in all other cases of GPU leaks, the emissions have been assumed to be level to the sensor. To ensure accurate determination of ζ(*z_s_*,*H*,*σ_v,z_∙d*), the vertical dispersion parameter *σ_v,z_* = 0.28 has been numerically selected to maximize the linear correlation (R^2^ = 0.74) between the estimated *Q_c_* and known *Q_m_*, and our results show that all the experimental cases of Table 1 in Figure 9a lie within the 50% accuracy interval of ideal linearity. It is notable that the calculated value of *σ_v,z_* is relatively close to the Pasquill–Gifford stability class A, which is consistent with experimental measurement conditions [44] (strong direct solar irradiance, ~2 m/s average wind speed).

Deviations from known leak rates of our include (1) the slow and unstable wind speeds, (2) short distances (<10 m) under consideration, and (3) the stochastic nature of short term (<1 h) CH_4_ peak measurements which introduce a strong component of sampling error in the retrieved [CH_4_](***r_s_***) and impact source inversion accuracy. Nevertheless, despite the aforementioned limitations and non-ideal measurement conditions of our heuristically parametrized Gaussian plume model, we note that our model provides *Q_c_* estimates within 50% accuracy of *Q_m_* based on a reconstruction of the Gaussian profile of the plume as the wind vector varies around the source-detector LOS. This accuracy tolerance is consistent with analysis from literature [49], demonstrating our calculations to yield results reasonable expectations within the typical demonstrated accuracy range of Gaussian source inversion models.

In conjunction with source localization techniques, we envision the methods of Section 3.3 and Section 3.4 to be applied sequentially, where the AOA (or leak region, based on source-detector LOS intersection identified using two or more TDLAS sensors) may be first identified, followed by quantification methods using the above Gaussian plume model. For cases where greater accuracy is required, a more contemporaneous machine-learning based approach has been validated [38], with initial results demonstrating promise towards greater accuracy of quantification. However, larger training sets are required to provide a greater confidence in the physical basis and model generalizability of our decision-tree based regressor, and results will be presented in an upcoming publication.

## 4. Conclusions

In this paper, we demonstrate a portable TDLAS spectrometer for real-time CH_4_ monitoring, which targets the 2ν_3_ R(4) CH_4_ transition at 6057.1 cm^−1^ with NEA = 4.5 × 10^−6^ Hz^−1/2^ corresponding to 2.0 ppmv∙Hz^−1/2^ over a 5 cm open-path measurement. The sensor is capable of both high-frequency (up to 2 ms time-resolution) and real-time operation (2 s resolution, 50% acquisition duty cycle), with 2.1 s chamber response time. During field testing at the METEC facility (CSU), 5 control and 2 blind experiments were successfully performed, corresponding to a total of 6.3 h of field testing data. Over this period, CH_4_ measurement correlation with a proximally located customized MOX sensor yields reasonable concentration correlation (R^2^ = 0.74) over source rates spanning 4.4 SCFH to 135 SCH, thus demonstrating our TDLAS sensor as a viable alternative to conventional chemical sensors, whilst providing additional species selectivity capability through targeting appropriate cross-talk free molecular transitions. AOA calculations are successfully demonstrated using a weighted average determination of leak-angle, which adequately identifies all leaks in both control and blind experiment scenarios and may be generalized for leak region identification using two or more TDLAS sensors. To determine the leak rate, we have demonstrated a parameterized Gaussian plume model for source magnitude estimation. Our data demonstrates good linearity between calculated and measured flow rates (R^2^ = 0.74), with vertical dispersion parameter *σ_z_* = 0.28, in reasonable agreement with expected values from literature. Given the non-idealities present in our deployment however, the Gaussian plume model yields an accuracy only slightly better than 50% for our control and blind experiments, and further improvement has been sought through the application of a random-forest regressor to demonstrate the efficacy of machine-learning based leak quantification [38]. As a concluding note, a second-generation integrated photonic chip sensor is currently under development [31,36] based on evanescent waveguide TDLAS of ambient CH_4_ [18,27]. Full integration of the source, detector, and sensing components on a single chip [50,51,52,53] will provide the same functionality of our free-space optical sensor, with the added benefit of miniaturization for superior SWaP-C performance. We envision the scalable nature of our integrated TDLAS sensing platform to enable a new generation of optical sensor networks for real-time, wide-area monitoring of trace-gas analytes. Preparations for a second field deployment at the METEC facility using prototype photonic chip sensors are currently underway, with results to be presented in an upcoming publication.

## Figures and Tables

**Table 1:** Summary of leak experiments at the METEC facility, with calculated leak angle-of-arrival (AOA) (0° corresponds to North). A total of 6.3 h of leaks from 3 well pads are performed, comprising of 5 control leaks of large magnitude (68 SCFH and above) and 2 blind leaks of smaller magnitude. Control experiments 1 and 4 are split into two flow rates of equal duration, and the two leak location angles in Controls 2 and 4 indicate simultaneous dual-leak points during the experiment.

Experiment	Leak Duration	LeakC		Flow Rate (SCFH)	Leak Location [Distance, Angle]	Average Wind-Velocity [Speed, Angle]	Leak AOA $\langle \alpha_{AOA}\rangle \pm \delta \alpha_{AOA}$
Control 1	3554 s	East GPU	(Pad 3)	68/127	9.0 m, (90°)	1.96 m/s, (137.2°)	102.3° ± 22.0°
Control 2	1757 s	West GPU	(Pad 3)	127	4.1 m, (90°,110°)	2.60 m/s, (125.6°)	109.9° ± 24.1°
Control 3	3553 s	East tank	(Pad 3)	135	13.0 m, (155°)	1.77 m/s, (116.8°)	145.5° ± 14.5°
Control 4	3552 s	West GPU	(Pad 3)	68/130	4.1 m, (90°,110°)	1.58 m/s, (97.0°)	102.3° ± 23.2°
Control 5	3459 s	East GPU	(Pad 3)	125	9.0 m, (90°)	2.80 m/s, (61.6°)	82.6° ± 13.2°
Blind 1	3442 s	Tank	(Pad 1)	36.1	3.4 m, (60°)	0.58 m/s, (68.9°)	76.3° ± 53.6°
Blind 2	3470 s	Wellhead	(Pad 2)	4.4	6.9 m, (125°)	1.41 m/s, (124.8°)	119.3° ± 25.2°
